# Mechanical, Morphological, Thermal and the Attenuation Properties of Heavy Mortars Doped with Nanoparticles for Gamma-Ray Shielding Applications

**DOI:** 10.3390/ma16083255

**Published:** 2023-04-20

**Authors:** Mohammed Thamer Alresheedi, Mohamed Elsafi, Yosef T. Aladadi, Ahmad Fauzi Abas, Abdullrahman Bin Ganam, M. I. Sayyed, Mohd Adzir Mahdi

**Affiliations:** 1 Department of Electrical Engineering, King Saud University, P.O. Box 800, Riyadh 11421, Saudi Arabia; 2 Physics Department, Faculty of Science, Alexandria University, Alexandria 21511, Egypt; mohamedelsafi68@gmail.com; 3 Department of Physics, Faculty of Science, Isra University, Amman 11622, Jordan; 4 Department of Nuclear Medicine Research, Institute for Research and Medical Consultations (IRMC), Imam Abdulrahman bin Faisal University (IAU), P.O. Box 1982, Dammam 31441, Saudi Arabia; 5 Wireless and Photonics Research Centre, Universiti Putra Malaysia, Serdang 43400, Selangor, Malaysia

**Keywords:** mortars, nanoparticles, gamma-rays, shielding, waste marble, stress–strain curve, transmission factor

## Abstract

This study aimed to develop a mortar composite with improved gamma ray shielding properties using WO_3_ and Bi_2_O_3_ nanoparticles, as well as granite residue as a partial replacement of sand. The physical properties and effects of sand substitution and nanoparticle addition on the mortar composite were analyzed. TEM analysis confirmed the size of Bi_2_O_3_ and WO_3_ NPs to be 40 ± 5 nm and 35 ± 2 nm, respectively. SEM images showed that increasing the percentage of granite residues and nanoparticles improved the homogeneity of the mixture and decreased the percentage of voids. TGA analysis indicated that the thermal properties of the material improved with the increase in nanoparticles, without decreasing the material weight at higher temperatures. The linear attenuation coefficients were reported and we found that the LAC value at 0.06 MeV increases by a factor of 2.47 when adding Bi_2_O_3_, while it is enhanced by a factor of 1.12 at 0.662 MeV. From the LAC data, the incorporation of Bi_2_O_3_ nanoparticles can greatly affect the LAC at low energies, and still have a small but noticeable effect at higher energies. The addition of Bi_2_O_3_ nanoparticles into the mortars led to a decrease in the half value layer, resulting in excellent shielding properties against gamma rays. The mean free path of the mortars was found to increase with increasing photon energy, but the addition of Bi_2_O_3_ led to a decrease in MFP and better attenuation, making the CGN-20 mortar the most ideal in terms of shielding ability among the prepared mortars. Our findings on the improved gamma ray shielding properties of the developed mortar composite have promising implications for radiation shielding applications and granite waste recycling.

## 1. Introduction

Due to advances in radiation applications, an increasing number of technologies use high energy photons to function. Ionizing radiation, which is a form of radiation that has sufficient energy to detach electrons from atoms, can cause long-term damage to the environment and to the human body. To avoid this damage, radiation shielding materials are designed to absorb as much radiation as possible for a specific application [1,2,3,4]. The material is specifically tailored for the application, which can vary in the type of radiation, the energy of the photons, whether the radiation needs to be sealed away completely or absorbed, etc. Because of this, certain materials are more suited depending on these factors than others, which leads to a wide range of radiation shielding materials being produced [5,6,7].

Cement mortar is a mixture of sand, water, and cement or lime as a binder that acts as a workable paste to bind building blocks to each other. Mortars are an essential radiation shield to construct the walls of radiotherapy rooms, nuclear reactors, and similar buildings. Mortar and concrete can also be a cheaper and more environmentally friendly alternative to ordinary Portland cement [8,9,10]. By itself, mortar has poor strength at ambient temperatures. However, by introducing particles into the mixture, these properties as well as the radiation shielding properties of the mortar can be enhanced. These mixes are used to attenuate both gamma-rays and neutrons, depending on the particles introduced. If heavy metal oxides (HMOs) are added, the density of the mortar will increase, enhancing the radiation absorption capability of the materials. Meanwhile, since these additives typically do not have a high neutron capture cross-section, their neutron shielding ability does not improve by the same amount, which is why it is useful to know the specifics of the application when designing the shielding material [11,12,13].

Currently, nanomaterials are used in a wide array of applications, including sensors, display technology, agriculture, medical drug delivery, solar cells, energy storage devices, and many others. More studies need to research the usefulness, characteristics, and applications of nanoparticles in radiation shielding, as they change depending on the synthesis process, temperature, pore size, pH, strain, and particle size when preparing the material [14,15,16,17]. Using nanoparticles as opposed to microparticles can especially be effective at enhancing the characteristics of a shielding material because nanoparticles can be distributed into the mix more evenly, leading to greater attenuation. Bismuth oxide is one such example of HMO nanoparticles that can improve the mechanical and gamma-ray shielding properties of concretes, which further improved as more bismuth oxide was added into the samples [18,19]. Different nanoparticles offer unique characteristics that can be tailored to enhance the thermal, mechanical, and radiation shielding properties of ordinary Portland cement pastes. For example, Fe_2_O_3_ and ZnO nanoparticles have been shown to improve the mechanical and radiation shielding properties of cement pastes [20,21]. Meanwhile, tungsten carbide, which has a high atomic number and excellent shielding ability, has been proposed as a replacement for lead, which was previously the industry standard for radiation shielding [22]. Studies have shown that tungsten carbide can improve the radiation shielding properties of cement pastes, while also offering greater resistance and hardness [23]. Pioneering studies in this field include the work of Smith et al. [14], who investigated the use of bismuth oxide nanoparticles for radiation shielding, and the study by Lee et al. [15], who explored the use of Fe_3_O_4_ nanoparticles for improving the radiation shielding properties of cement pastes. These studies, and others like them, have greatly contributed to the current understanding of the use of different nanoparticles for radiation shielding in various applications. From these previous studies, the design and development of radiation shielding materials that are both effective and efficient is absolutely necessary in order to reduce the potential for long-term harm to human health and the environment. The incorporation of nanomaterials into these shielding materials presents exciting new possibilities for improving their attenuation capacities.

In this work, different mortar samples with Bi_2_O_3_ and WO_3_ nanoparticles were prepared to investigate their thermal, mechanical, and morphological as well as the radiation shielding properties at a wide range of energies. Despite the fact that earlier research on radiation shielding materials has produced some encouraging findings, there are still some limitations and obstacles that need to be solved. While our study aims to investigate the effect of particle size on the radiation shielding properties of red clay mixed waste marble, it is important to note that our findings may be limited by the sample size and the specific materials used. Nevertheless, our study may provide valuable insights into the potential of red clay mixed waste marble as a radiation shielding material and could inform future research in this area.

## 2. Materials and Methods

The binder in this work is CEM I-52.5N Portland cement with Blaine fineness characteristics of 410 m^2^/kg, specific gravity of 3.13, and the compressive strength after 7 days is 65 MPs. Ordinary sand was a fine aggregate, and the grain size of the sand was standard for use in the mortar composition. The raw sand was obtained through the process of correcting the grain size by sifting, and drying was carried out to remove all the moisture present in the sand. Granite residue used for partial replacement of sand was obtained from the process of polishing and cutting granite blocks from one of the granite production factories. The granite residue was carefully collected and transported in wet form. Before use, the residue was sifted and homogenized to ensure uniformity. WO_3_ and Bi_2_O_3_ NPs were prepared chemically by coprecipitation method [11,24] and used as fine aggregates added to the mortar composite to show the improvement of gamma-ray shielding properties, as with the addition of these particles, the density of the slurry composite increases. The ratio of the prepared mortar mixture to other studies aimed at improving the radiation shielding ability [25,26,27]. This mixture was calculated according to the volume by replacing part of the granite dust with sand and the addition of a small amount of nanoparticles in the types of prepared mortars. This resulted in sand substitution ratios of 0, 10, 20, 30, and 40%, as well as the addition of nanoparticles in the same proportion from cement, as can be seen in Table 1. Before preparing the mortar, the physical properties of the materials used were determined, where sand and granite aggregates were sifted with a 60-micrometer sieve; in addition to determining the compositions in each material, EDX analysis (energy dispersive X-ray, JEOL ltd: JMS-IT 200, accelerated voltage 20 keV) [28] was used as shown in Table 2. Furthermore, the size of the nanoparticles added to the mortar was determined using TEM (high transmission electron microscope, JEOL JEM-2100, accelerated voltage 200 keV) [29].

**Figure 1:**
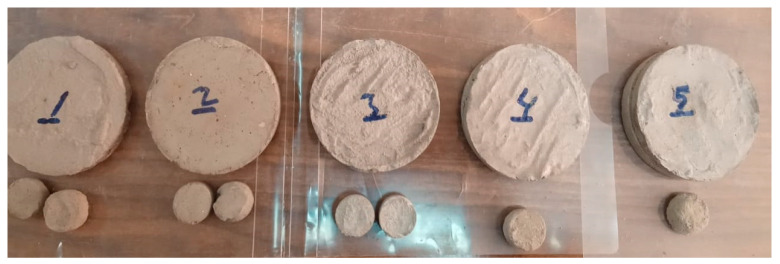
Picture of real prepared samples.

Mortar samples were prepared in a controlled and well-ventilated laboratory, where the preparation was performed through the casting process [30] and the samples were left to dry and harden for two weeks. The image of different prepared mixtures of mortars is shown in Figure 1.

**Figure 2:**
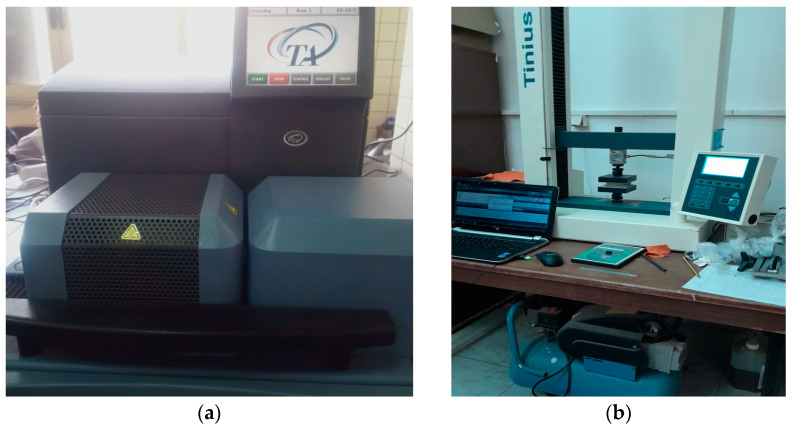
(**a**) Thermal analysis, (**b**) generic compression devices used in this work.

After preparing the mixtures, a thermal analysis device, as shown in Figure 2a, was used to calculate the weight loss by increasing the temperature or TGA-analysis (TGA-55, New York, NY, USA). The stress–strain curve was also determined using a mortar sample of 10 mm thickness and 80 mm diameter on a generic compression stress vs. strain device (Tinius Olsen, 10 kN), as shown in Figure 2b.

**Figure 3:**
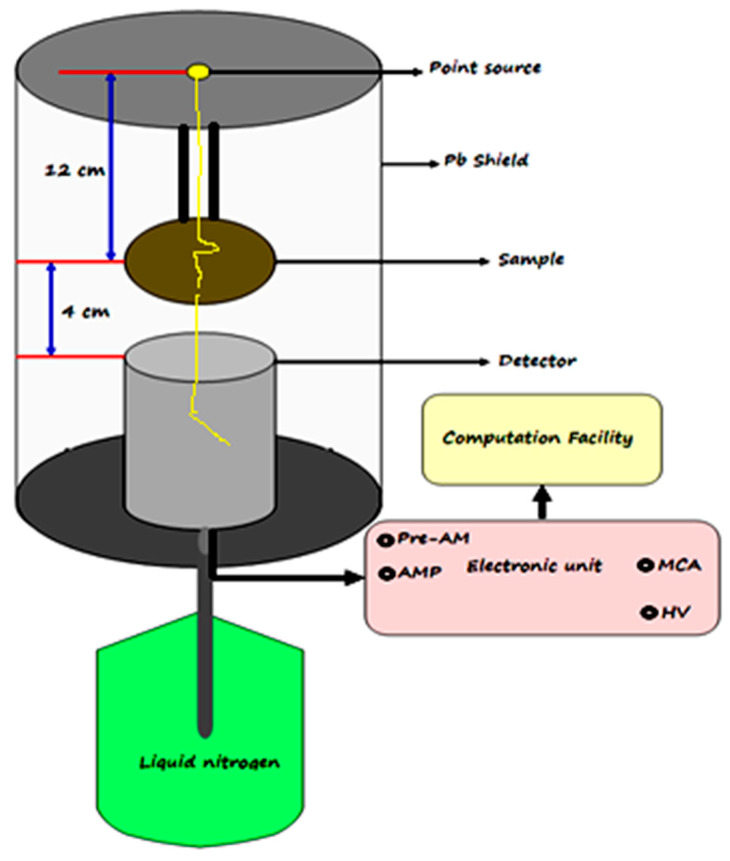
The geometry of the experimental gamma-rays attenuation measurements.

SEM images of the prepared mortars were also studied using a JEOL Ltd: JMS-IT 200 scanning electron microscope, where a cross section of each sample was taken and visualized at a magnification factor of 2000 and an acceleration voltage of 20 keV. For gamma-ray attenuation measurements, amriciam-241 (has an energy line of 0.060 MeV), cesium-137 (has an energy line of 0.662 MeV), and cobalt-60 (has two energy lines, 1.173 and 1.333 MeV) point sources were used as radiated gamma lines as well as a high-purity germanium (HPGe) detector with 1.92 keV energy resolution at 1.333 keV cobalt line and relative efficiency of 24% was used to detect and translate incident photons into electrons. The geometry of the experimental gamma-ray attenuation measurements in the current work is shown in Figure 3, where the source was placed vertically through the sample to the detector with the help of a collimator to obtain the incident narrow beam.

After the detector was calibrated [31], the produced spectrum in the absence of a sample or its presence was analyzed using Genie-2000 software to obtain the count rate or the area formed under the resulting peak of photon energy in both cases with the same conditions. From the evaluation area within (A) and without (A_0_) the mortar sample of thickness (t), the linear-attenuation coefficient (LAC) can be determined by [32]:

(1) $$LAC\;\left({\mathrm{cm}}^{-1}\right)=\frac{1}{t\;\left(\mathrm{cm}\right)}\;\ln\;\frac{A_{0}}{A}\;$$

The transmission factor (TF) represents the intensity ratio in both cases at a certain absorber distance and is given by the following formula [33]:

(2) $$TF\;\left(\%\right)=\frac{I}{I_{0}}\times 100=\frac{A}{A_{0}}\times 100$$

The other attenuation factors based on LAC calculation, such as the half value layer (HVL), mean free path (MFP), and tenth value layer (TVL), can be estimated from the following equation [34,35]:

(3) $$HVL=\frac{\ln2}{LAC}\;,\;MFP=\frac{\ln2}{LAC}\;,\;TVL=\frac{\ln10}{LAC}\;$$

The efficiency of shielding mortar samples was estimated using an important parameter called the radiation protection efficiency (RPE) and was calculated by the following equation [36]:

(4) $$HRPE\left(\%\right)=\left[1-\frac{A}{A_{0}}\right]\times 100\;$$

## 3. Results and Discussion

### 3.1. TEM and SEM Results

**Figure 4:**
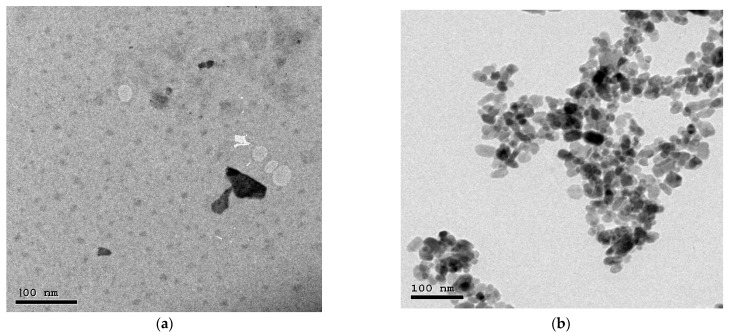
TEM images of (**a**) Bi_2_O_3_ NPs and (**b**) WO_3_ NPs.

**Figure 5:**
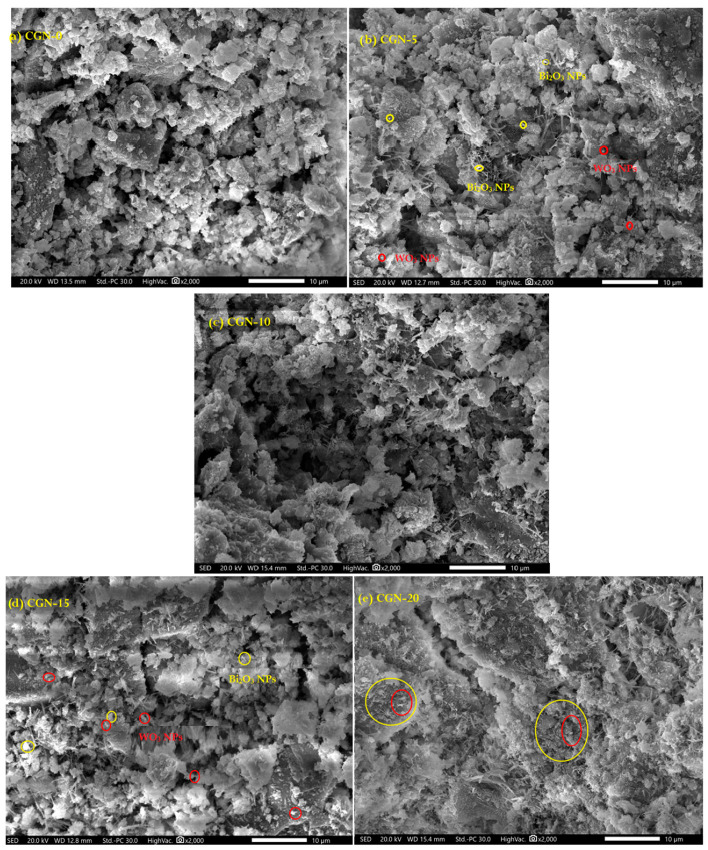
SEM images of prepared mortars, (**a**) CGN-0, (**b**) CGN-5, (**c**) CGN-10, (**d**) CGN-15, and (**e**) CGN-20.

First, the nanopowder was photographed by transmission electron microscope to verify its size as shown in Figure 4, and it turned out that the average size of the Bi_2_O_3_ NPs was 40 ± 5 nm, while the average size of the WO_3_ NPs was 35 ± 2 nm. The five prepared mortar mixtures were imaged by SEM technique as shown in Figure 5. It is clear from the results that with the increase in the percentage of granite residues replaced by sand and nanoparticles in the mixture, the lower the percentage of voids in the mixture and the homogeneity in the mixture is better.

### 3.2. Thermal Results

**Figure 6:**
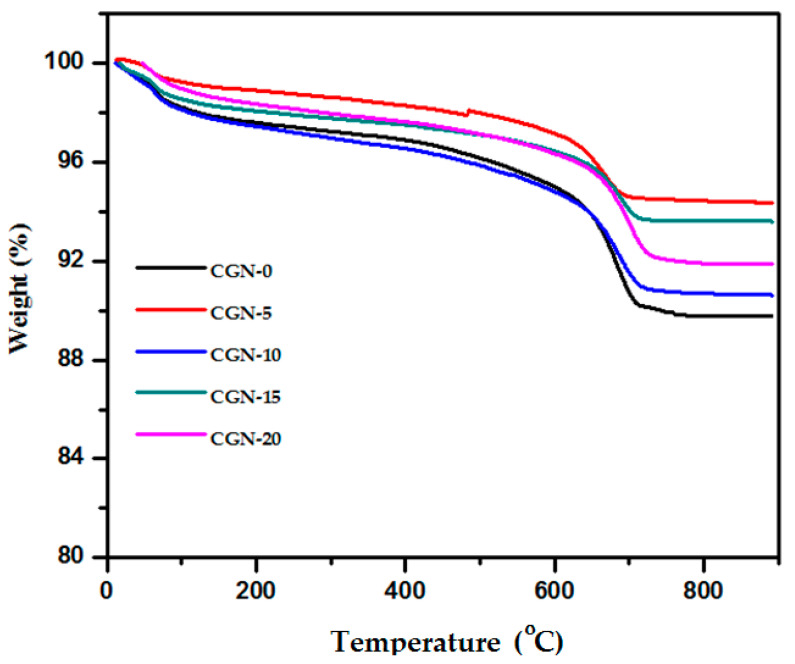
TGA of prepared mortar samples.

Thermal gravimetric analysis (TGA) was used for the prepared mortars and the results are shown in Figure 6, where the weight loss was taken as a function of temperature. The results display the weight loss due to the influence of temperature, where at 200 °C, the weight losses were 2.11, 1.38, 2.16, 1.83, and 1.65% for CGN-0, CGN-5, CGN-10, CGN-15, and CGN-20, respectively. With the increase in temperature, a small gradient of decreases is observed till approximately 600 °C and the percentages of weight losses were 2.96, 2.11, 2.94, 1.93, and 1.95%, respectively. After 600 °C, there was a rapid decline and then stability to keep the remaining weights of each mortar studied after exposure to 900 °C as follows: 89.30, 94.35, 90.63, 93.60, and 91.89% for CGN-0, CGN-5, CGN-10, CGN-15, and CGN-20, respectively. From these results, it is clear that the increase in nanoparticles does not decrease the weight of the material with the increase in temperature, but rather improves the thermal properties of the material.

### 3.3. Mechanical Results

**Figure 7:**
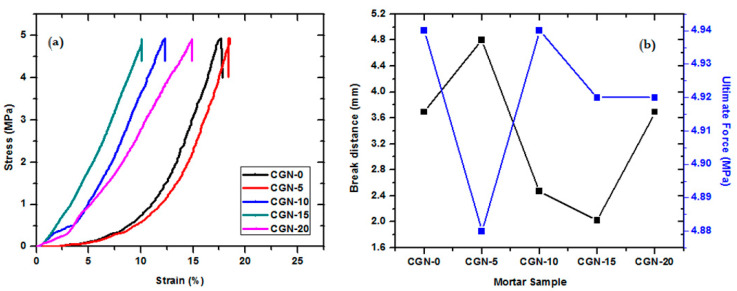
(**a**) The stress–strain curve, (**b**) the break distance and ultimate force of prepared samples.

The stress–strain curve was determined for all five prepared mortar samples as shown in Figure 7a, and the break distance as well as the ultimate force of the prepared samples were estimated as shown in Figure 7b. The break distances were 3.69, 4.8, 2.47, 2.02, and 3.00 mm for CGN-0, CGN-5, CGN-10, CGN-15, and CGN-20, respectively. The second sample (CGN-5) has more compressive stress than the control mortar (CGN-0) and the CGN-20 is better than the CGN-10 and CGN-15. The results indicated that the addition of nanoparticles with the addition of granite dust does not negatively affect its mechanical properties, and therefore it can be used as a protective material against radiation.

### 3.4. Gamma-Rays Attenuation Results

**Figure 8:**
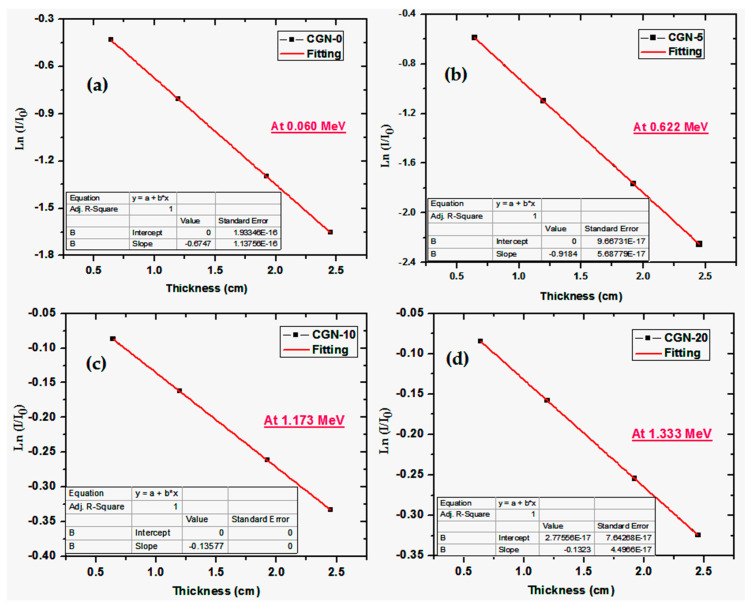
The relation between Ln (I/I_0_) and the mortar thickness. (**a**) For CGN-0 sample at 0.060 MeV, (**b**) for CGN-5 sample at 0.662 MeV, (**c**) for CGN-10 sample at 1.173 MeV, and (**d**) for CGN-20 sample at 1.333 MeV.

Figure 8 shows the relationship between Ln (I/I_0_) and mortar thickness for each of the samples at a specific energy. (a) For the CGN-0 sample at 0.060 MeV, (b) for the CGN-5 sample at 0.662 MeV, (c) for the CGN-10 sample at 1.173 MeV, and (d) for the CGN-20 sample at 1.333 MeV. From the Lambert–Beer law: Ln (I/I_0_) = −µ x, which means that the slopes in each of the subfigures represent the linear attenuation coefficient, represented as µ or LAC.

The slope at all four energies and for each of the prepared mortars is negative, which means that the transmission of the photons through the samples decreases when increasing the mortars. In other words, for a mortar sample of small thickness, such as 0.641 cm, this transmission factor, or TF, is at its highest, but as the thickness of the mortar increases to 1.196 cm, TF decreases, which means that the samples can attenuate more photons than the same mortar of smaller thickness. Further increasing the thickness of the mortars to 1.924 cm leads to the samples attenuating even more photons than at thicknesses of 0.641 and 1.196 cm. Therefore, this figure represents the LAC of the samples and illustrates the impact that the thickness of the sample has on its attenuation performance.

**Figure 9:**
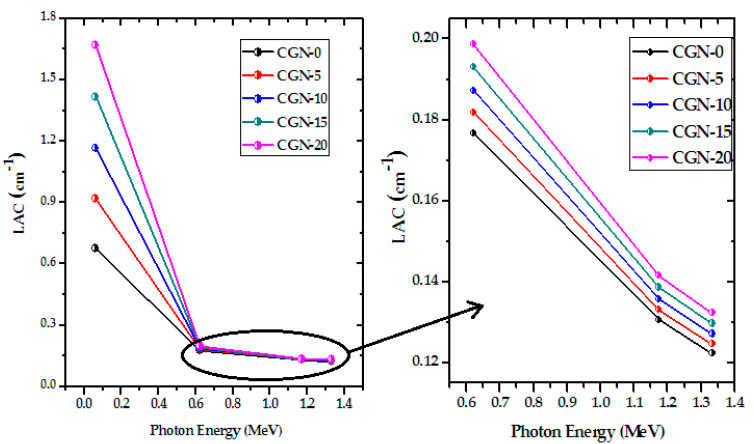
The LAC as a function of energy for different prepared CGN-mortar samples.

The LAC of the prepared CGN mortars is plotted as a function of energy in Figure 9. From the figure, LAC exponentially decreases with energy, which agrees with the Lambert–Beer law. The LAC values start at their maximum and decrease to a minimum at 1.333 MeV. This trend means that the attenuation capability of the mortars is at its best against photons with energies of 0.06 MeV and decreases as the energy of the photons increases up to 1.333 MeV. The figure also demonstrates the effect of introducing Bi_2_O_3_ nanoparticles into the mortar. This additive increases the LAC values at all tested energies, and is most evident at the first energy, where the photoelectric effect is most dominant. The probability for photoelectric effect to occur is highly dependent on the atomic number of the absorber. Since Bi has a high atomic number, as more Bi_2_O_3_ was added into the mortar, LAC increases. It is important to mention that nanoparticles have a larger surface area to volume ratio, which leads to better distribution in the mixture and a higher probability of interaction with radiation. Moreover, the small size of nanoparticles allows for a more effective packing and arrangement of particles, thus providing better radiation absorption capability. At 0.06 MeV, the LAC value increases by a factor of 2.47 when adding Bi_2_O_3_, while it is enhanced by a factor of 1.12 at 0.662 MeV. Thus, the incorporation of Bi_2_O_3_ nanoparticles can greatly affect the LAC at low energies, and still have a small but noticeable effect at higher energies.

**Figure 10:**
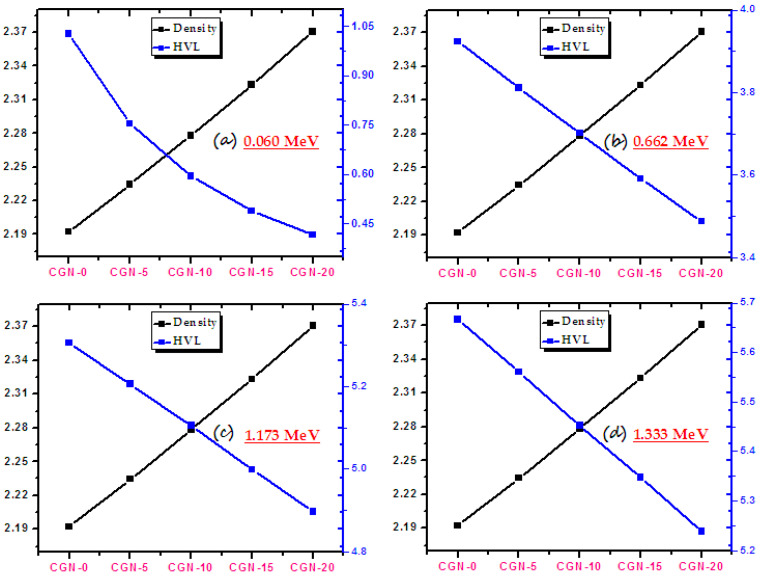
The HVLs and the densities of prepared CGN-samples at different energies. (**a**) at 0.060 MeV, (**b**) 0.622 MeV, (**c**) 1.173 MeV and (**d**) 1.333 MeV.

Figure 10 demonstrates the relationship between the HVL and the density of the prepared mortars. At any of the examined energies, density has an inverse relationship with HVL. In other words, when Bi_2_O_3_ was added into the mortars, its density increased while its HVL decreased. This behavior can be explained as follows. In mortars where the percentage of Bi_2_O_3_ nanoparticles is large, meaning that they have a high density, the atoms are very close to each other. Therefore, when a photon enters the mortar, the possibility of it interacting with the atoms of the mortars is large. Because of this, most of the photons are absorbed or scattered, while only a small number of these penetrate from the front side of the mortar to the back, which means that the sample has excellent shielding properties.

**Figure 11:**
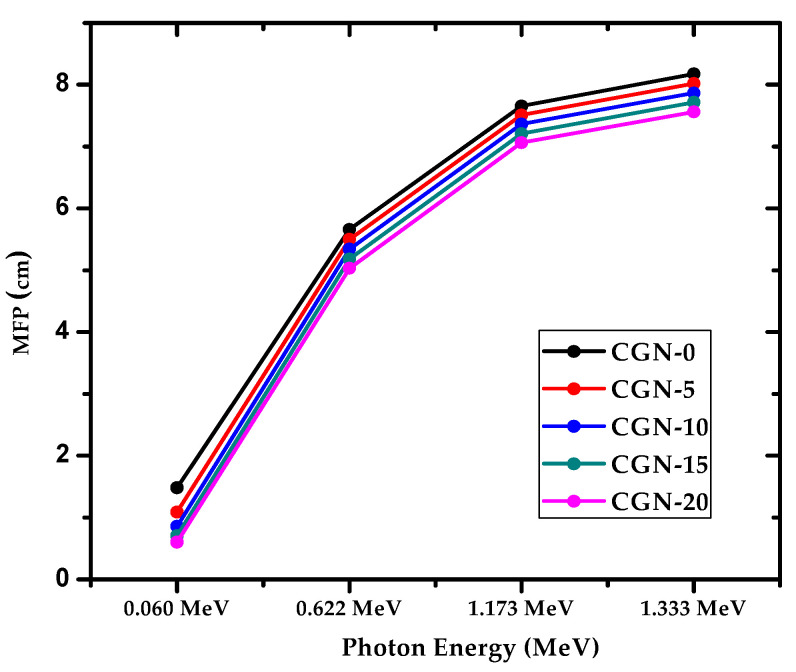
The MFP of prepared CGN-samples at different energies.

The mean free path, or MFP, of the mortars is graphed in Figure 11 against photon energy. The minimum MFP values are observed at the lowest tested energy, 0.06 MeV, and increase to a maximum at 1.333 MeV, for all the prepared samples. For example, CGN-5’s MFP starts at 1.089 cm at 0.06 MeV, and increases to 5.500 cm at 0.622 MeV, 7.511 at 1.173 MeV, and 8.022 at 1.333 MeV. Meanwhile, CGN-15’s MFP is equal to 0.0706, 5.180, 7.211, and 7.715 cm for the same respective energies. Because higher energy photons lead to an increase in the MFP of the mortars, less photons are attenuated at higher energies, and thus less shielding is provided. One way to improve the MFP values at any energy is to increase the Bi_2_O_3_ content in the mortars. At 1.173 MeV, CGN-0, the sample with no Bi_2_O_3_, has the smallest MFP equal to 7.654 cm. This value decreases to 7.511 cm for CGN-5, 7.366 cm for CGN-10, 7.211 cm for CGN-15, and 7.065 cm for CGN-20. Therefore, increasing the Bi_2_O_3_ content in the samples causes the distance between subsequent collisions to decrease, which leads to better attenuation. In other words, the CGN-20 mortar has the most ideal shielding ability of the prepared mortars.

**Figure 12:**
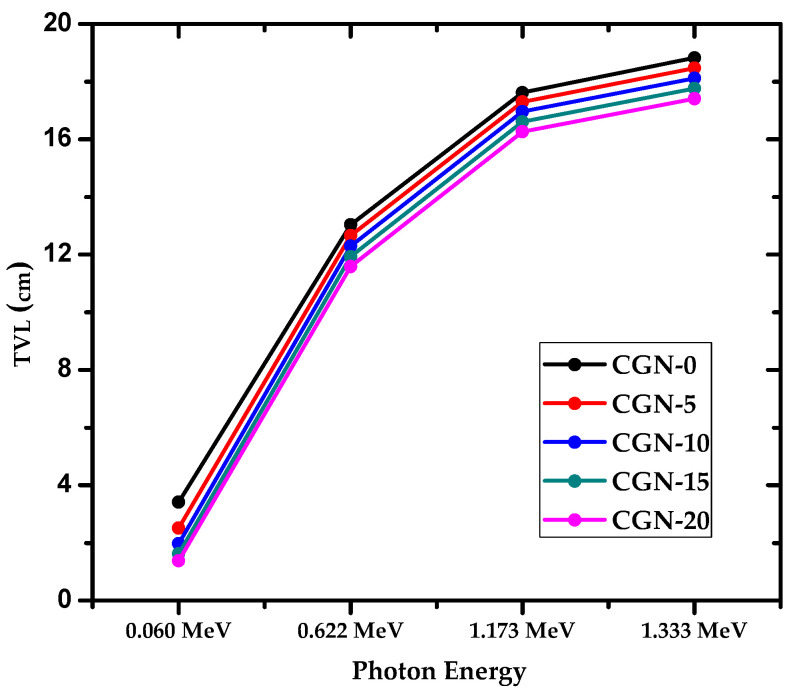
The TVL of prepared CGN-samples at different energies.

Figure 12 shows the tenth value layer, or TVL, of the mortars as a function of the incoming photon energy. Since TVL represents the thickness of the sample needed to reduce the amount of incoming radiation to a tenth of its original value, a smaller value denotes that a thinner sample is needed, which is ideal to limit the space that the shield needs. At all four energies, CGN-0 has the greatest TVL, while CGN-20 has the smallest TVL. For instance, at 0.06 MeV, CGN-0 has a TVL of 3.413 cm while CGN-20 has a TVL of 1.381 cm, while at 1.333 MeV the TVLs are equal to 18.823 cm and 17.404 cm for CGN-0 and CGN-20, respectively. This result means that increasing the Bi_2_O_3_ content in the samples decreases the thickness required to attenuate nine-tenths of the incoming photons, making CGN-20 more space efficient. The figure also shows that if the energy of the photons increases, then a thicker shield is needed to provide the same level of attenuation. For example, CGN-10’s TVL is equal to 1.977 cm at 0.06 MeV, but increases to 12.297 cm at 0.622 MeV, 16.960 cm at 1.173 MeV, and 18.115 cm at 1.333 MeV. Therefore, the energy of the photons needs to be taken into account when determining the ideal thickness for a radiation shield in a specific application.

**Figure 13:**
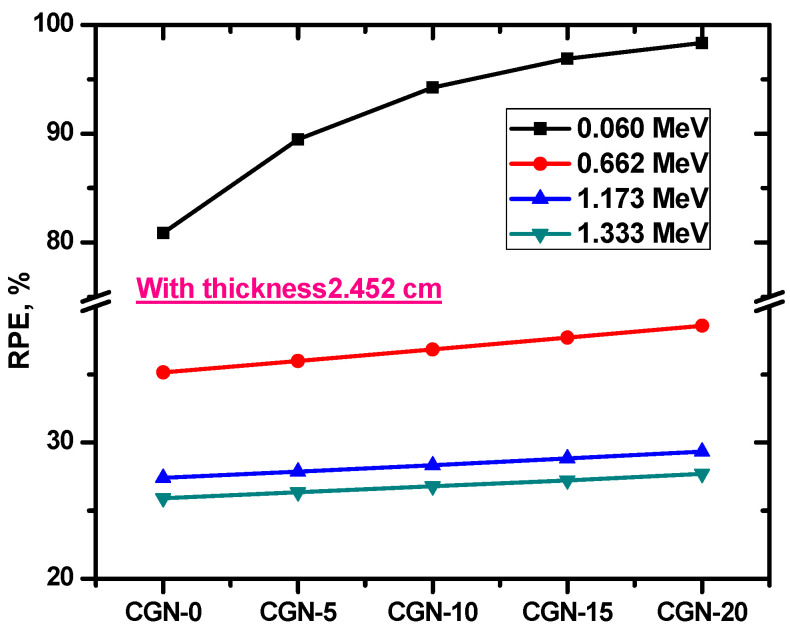
The radiation protection efficiency (RPE) for the prepared mortars with a thickness of 2.452 cm.

The radiation protection efficiency (RPE) for the prepared mortars with a thickness of 2.452 cm is presented in Figure 13. At 0.06 MeV, the RPE for CGN-0 is equal to 80.88%. Meanwhile, when the Bi_2_O_3_ content increases to 5%, the RPE rises to 89.48%, to 94.25% for 10% Bi_2_O_3_, and to 98.32% for 20% Bi_2_O_3_. Two conclusions can be made from these data. First, RPE increases due to the addition of Bi_2_O_3_, which means that the radiation protection capability of these mortars improves due to this additive. Second, the mortars can attenuate most of the incoming photons at 0.06 MeV, especially CGN-10, 15, and 20. However, as the incoming photon energy increases, RPE decreases, which means that the shielding ability of the mortars worsens when the samples are exposed to radiation with energy greater than 0.662 MeV.

## 4. Conclusions

To summarize, this study investigated the effects of incorporating Bi_2_O_3_ and WO_3_ nanoparticles on the properties of mortar composites. The results showed that the addition of nanoparticles improved the homogeneity and reduced voids in the prepared mortars, resulting in improved thermal stability, mechanical strength, and radiation shielding capabilities. The addition of Bi_2_O_3_ nanoparticles did not negatively affect the mechanical properties of the CGN mortars, with the ultimate force of CGN-5 estimated to be higher than CGN-0, and CGN-20 having a better ultimate force than CGN-10 and CGN-15. Break distances for the samples ranged from 2.02 to 4.8 mm, with CGN-5 having the highest value and CGN-0 having the lowest. Besides, our study has demonstrated that the addition of Bi_2_O_3_ nanoparticles to CGN mortars can significantly enhance their radiation shielding properties. Specifically, we found that the LAC values of the mortars increased by up to 2.47 times at 0.06 MeV and 1.12 times at 0.662 MeV, while their density increased and HVL decreased. Additionally, the incorporation of Bi_2_O_3_ nanoparticles can improve the MFP and TVL of the mortars, with the MFP values of the CGN-20 sample decreasing from 7.654 to 7.065 cm at 1.173 MeV, and the TVL values of the CGN-20 sample decreasing from 18.823 to 17.404 cm at 1.333 MeV.

## Figures and Tables

**Table 1:** Mass composition of the prepared mortars.

Mortar-Code	Cement (g)	Granite Dust (g)	Sand (g)	Bi_2_O_3_ NPs	WO_3_ NPs	w/c Ratio
CGN-0	100.00	0.00	681.00	0.00	0.00	0.49
CGN-5	100.00	68.10	612.90	5.00	5.00	0.50
CGN-10	100.00	136.20	544.80	10.00	10.00	0.52
CGN-15	100.00	204.30	476.70	15.00	15.00	0.52
CGN-20	100.00	272.40	408.60	20.00	20.00	0.53

**Table 2:** Oxides percentage for the raw materials used.

Oxide Composition (%)	CEM I-52.5 N	Granite Dust	Sand
CaO	61.39	1.43	2.32
SiO_2_	19.55	60.67	91.61
Al_2_O_3_	4.82	11.12	2.92
Fe_2_O_3_	3.52	3.08	1.71
MgO	1.83	1.59	0.70
SO_3_	2.76	2.11	0.00
K_2_O	0.74	2.02	0.51
Na_2_O	0.14	2.14	0.50
L.I.O	2.18	3.40	1.72

## Data Availability

All data generated or analyzed during this study are included in this published article.
